# Rare ileal localisation of angiolipoma presenting as chronic haemorrhage and severe anaemia: a case report

**DOI:** 10.1186/1752-1947-2-129

**Published:** 2008-04-29

**Authors:** Nicola Della Volpe, Luigi Bianco, Claudio Bonuso, Mario Annecchiarico, Pierino Di Silverio, Assunta Caiazza

**Affiliations:** 1 Department of General and Geriatric Surgery, Diagnostic and Operative Endoscopy, School of Medicine, University of Naples 'Federico II', Italy

## Abstract

**Introduction:**

Angiolipomas are frequently observed benign tumours. They have a typical vascular component and are often located in subcutaneous tissues, and more rarely, in the gastrointestinal tract.

**Case presentation:**

We report an uncommon case of an angiolipoma found in the lower portion of the small bowel of an 80-year-old man who was undergoing evaluation for chronic anaemia. A standardised diagnostic pathway was followed. Endoscopic and radiological findings were negative. The diagnosis was finally established with the aid of capsule endoscopy. The case we report is the first in the literature of an angiolipoma without specific painful symptoms. In fact, the patient did not complain of abdominal pain or alvus changes, and abdominal examination did not suggest an expansive process. The endoscopic study performed with the capsule identified the lesion as the cause of the ingravescent anaemia. Intra-operative histological examination of the lesion made it possible to avoid a major surgical procedure and assured a short postoperative course for the patient.

**Conclusion:**

This report focuses on the importance of correct pre- and/or intra-operative histological diagnosis in order to offer the best therapeutic choice. An angiolipoma was suspected in this case, even though they are rarely located in the ileum.

## Introduction

Angiolipomas are benign tumours consisting of typical proliferative vascular tissue. They are generally located in subcutaneous tissues. The areas most involved include the upper and lower limbs and the trunk, while gastrointestinal localisation is extremely rare [1,2].

In our opinion, when angiolipoma is suspected, it is crucial to determine the diagnosis pre-operatively and, whenever possible, to clarify the diagnosis intra-operatively by histological examination, in order to perform the optimal procedure.

From a clinical point of view, this case involved an asymptomatic patient and because of this, the case report is novel.

## Case presentation

An 80-year-old man who underwent triple aortocoronary bypass surgery was affected by an aneurysm of the abdominal aorta, bilateral obstructive arteriopathy of the lower limbs, colonic diverticulosis, and chronic obstructive pulmonary disease. He came to our attention in June 2006 for evaluation of a normocytic hypochromic anaemia (Hb, 9.7 g/dl; haematocrit, 34%; sideremia, 28 mcg/dl). He did not complain of melena or haematemesis, although a faecal occult blood test was positive. The patient did not complain of abdominal pain or alvus changes, and abdominal examination did not suggest an expansive process. During hospitalisation, routine haematology and biochemical tests were repeated and anaemia was confirmed. Tumour markers (alpha-fetoprotein, carcinoembryonic antigen, tissue polypeptide specific antigen, carbohydrate antigen 19-9, cancer antigen 15-3, cancer antigen 125, prostate specific antigen) were negative, except for gastrin (155 pg/ml).

To identify the cause of the anaemia, oesophago-gastro-duodenoscopy was performed, which did not reveal any pathological finding, Anorectal-colonoscopy that confirmed diverticulosis only, with no signs of inflammation or past or present haemorrhage.

**Figure 1:**
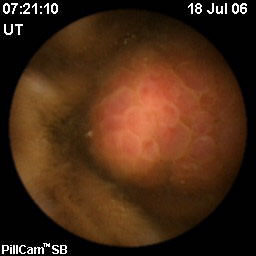
Ileal polypoid neoformation shown by capsule endoscopy.

A lesion located in the small bowel was suspected to be the cause of the anaemia, after an episode of enterorrhagia led to a fall in the patient's haemoglobin (Hb, 6.0 g/dl). Since a double balloon enteroscopy was not possible, a capsule endoscopy was performed. This investigation revealed the presence of a polypoid lesion located in the terminal ileal loop, occupying two-thirds of the lumen and covered by strongly congested and hypervascularised mucosa (Figure 1).

Abdominal pre- and post-contrast enhancement computed tomography scans showed only slight thickening and an anomalous impregnation of the mucous profile of the terminal ileal loop, with no loco-regional lymphadenopathy.

**Figure 2:**
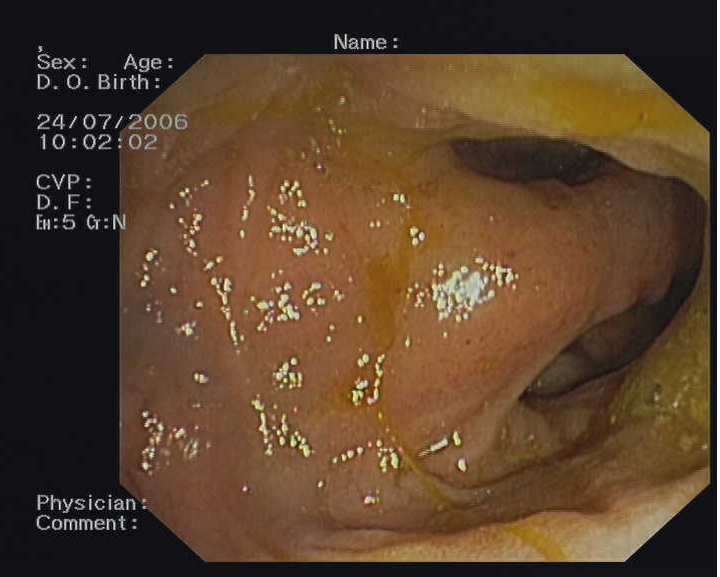
Ileal polypoid neoformation with a hypervascularised basement as it appeared during retrograde ileoscopy.

The patient underwent a second colonoscopy with a retrograde ileoscopy to obtain a tissue diagnosis of the lesion. This examination confirmed the presence of ileal sessile polypoid lesions with a hypervascularised basement, covered by a strongly congested mucosa (Figure 2). As it was not possible to carry out a biopsy of the lesion, the histological pre-operative diagnosis was not defined.

**Figure 3:**
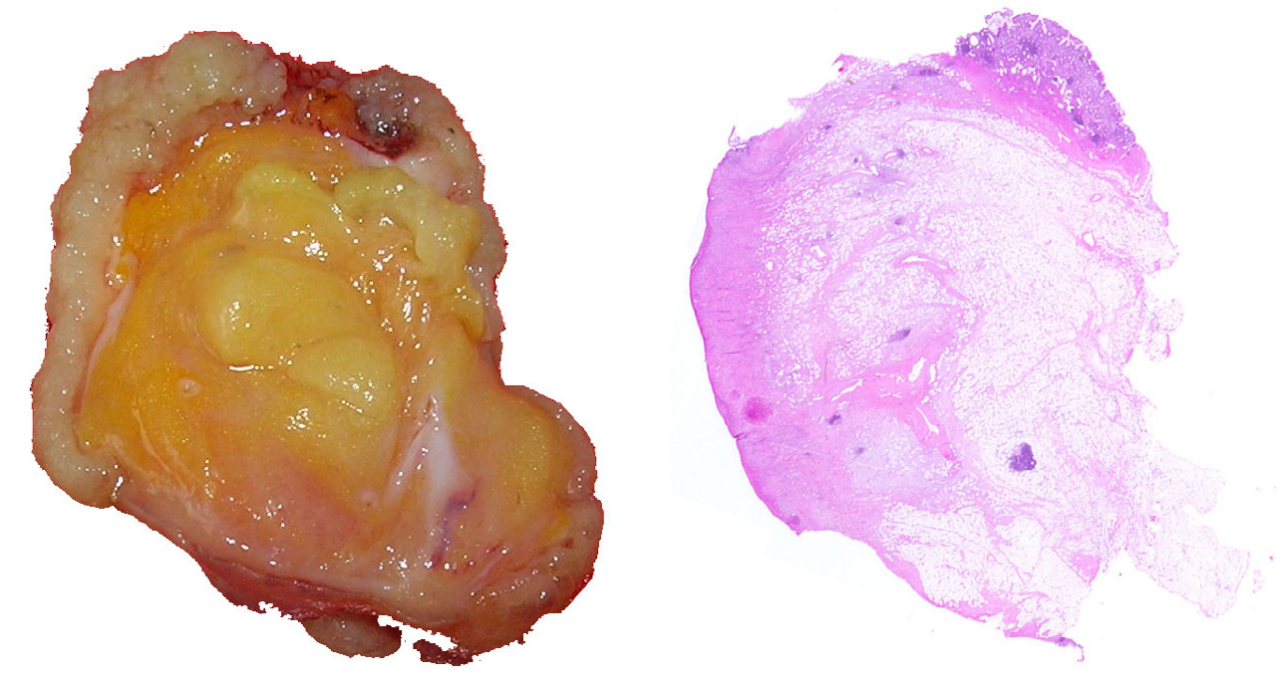
Specimen of the opened lesion and its microscopic appearance. Haematoxylin and eosin stain, magnification ×100.

Surgical exploration by laparotomy was performed. The respiratory insufficiency in our patient, with an American Society of Anesthesiologists status IV, meant laparoscopic surgery was contraindicated. An enterotomy was made in the terminal ileal loop about 30 to 40 cm from the ileocaecal valve. The polypoid lesion (2 × 1.5 cm^2^) was removed. Frozen section histological examination was conducted (Figure 3) and showed a submucosal lesion consisting of richly vascularised fibro-adipose tissue consistent with a lipoma and/or angiolipoma, and an ulceration on the mucosal surface with a fibro-inflammatory reaction, but without any cellular atypia.

It was not necessary to proceed to resection because the lesion was not malignant, even if there is no difference in risk between an enterotomy and small bowel resection. The surgical procedure was finished after careful haemostasis and enterorrhaphy.

The definitive histological diagnosis was a submucosal angiolipoma (Figure 3). The patient was discharged from the hospital on the fourth postoperative day.

## Discussion

Angiolipomas are benign tumours. They are encapsulated nodules consisting of mature adipose tissue, but are clearly different from lipomas because of the intralesional proliferation of vascular tissue. They usually affect young adults, most often as painful lesions located in the subcutaneous tissue of the limbs and trunk.

Angiolipomas of the gastrointestinal tract are uncommon. In a review of the literature, we found only 12 cases of angiolipomas involving the gastrointestinal tract. One angiolipoma was located in the oesophagus [3], three in the stomach [1,2,4], two in the duodenum [5,6], four in the colon [7,8], and two were at the ileocaecal valve. The small intestine was involved In only two cases [9,10].

This case is the third such report in the literature. However, it is the first case in which the patient did not exhibit any symptoms, such as pain or alvus alteration, but this patient did have chronic anaemia.

For this reason, the gastrointestinal tract was investigated by endoscopy. All examinations were negative, except for capsule endoscopy, which allowed us to identify and localise the lesion which was the cause of the ingravescent anaemia.

It was not possible carry out double balloon enteroscopy as we had no experience of performing this procedure on such a high-risk patient.

## Conclusion

In this case report, we have focused on the importance, whenever an angiolipoma is suspected, of a correct diagnosis made before or during surgery in order to afford the surgeon the opportunity to offer the patient the best treatment. In our case, the diagnosis of a benign lesion was made with the use of capsule endoscopy and successive retrograde ileoscopy, and confirmed by computed tomography scan. We think that in such a case, double balloon enteroscopy could also be useful to find suspected ileal lesions and to make an accurate pre-operative histological diagnosis. In this case, since it was not possible to make a pre-operative histological diagnosis, we performed histological examination during the surgical procedure. In this case we chose a conservative procedure rather than a resective one, even though there is no important difference between an enterotomy and small bowel resection.

We would like to conclude by emphasising the importance of a pre-operative focused and accurate diagnosis of the lesion, whenever angiolipoma is suspected, in order to choose the most appropriate surgical treatment. This can avoid a longer and sometimes more complicated postoperative course, and reduce medical costs as well.

## Competing interests

The authors declare that they have no competing interests.

## Authors' contributions

NDV, LB, CB, and AC performed the procedures (surgery and endoscopy). MA and PDS edited the report and compiled the reference list. All authors read and approved the final manuscript.

## Consent

Written informed consent was obtained from the patient for publication of this case report and accompanying images. A copy of the written consent is available for review by the Editor-in-Chief of this journal.
